# *Plasmodium* DNA ligase I is essential for parasite blood- and liver-stage development

**DOI:** 10.1128/msphere.00674-25

**Published:** 2025-12-15

**Authors:** Eisha Pandey, Shivani Mishra, Aastha Varshney, Saman Habib, Satish Mishra

**Affiliations:** 1 Division of Molecular Microbiology and Immunology, CSIR-Central Drug Research Institute 30082, Lucknow, India; 2 Academy of Scientific and Innovative Research (AcSIR) 550336, Ghaziabad, India; 3 Division of Biochemistry and Structural Biology, CSIR-Central Drug Research Institute 30082, Lucknow, India; Weill Cornell Medicine, New York, New York, USA

**Keywords:** *Plasmodium*, DNA ligase I, organelle genome, replication, liver stage

## Abstract

DNA ligases are a fundamental class of enzymes required for DNA replication and repair. They catalyze the formation of phosphodiester bonds, specifically at single-strand breaks in double-stranded DNA. The nuclear genome of malaria parasites encodes a single DNA ligase that is likely involved in nuclear and organellar DNA replication and repair. DNA ligase I from *Plasmodium falciparum* (*Pf*Lig1) has been biochemically characterized and shown to possess nick-sealing activity. However, its localization and function in the three genome-containing compartments—the nucleus, apicoplast, and mitochondrion—of the malaria parasites remain unknown. Here, we found that Lig1 is located primarily in the nucleus in both human and rodent malaria parasites throughout the parasite life cycle. Furthermore, we detected its presence in organelles via a chromatin immunoprecipitation-PCR assay. Our attempts to disrupt *Plasmodium berghei* Lig1 (*Pb*Lig1) in the blood stages have failed, indicating that the gene is likely essential. Next, we used an Flp/FRT-based conditional mutagenesis system that silences gene function in sporozoites. We demonstrated that *Pb*Lig1 is essential for parasite liver-stage development. Sporozoites lacking *Pb*Lig1 invade hepatocytes but arrest growth during mid-liver-stage development. *Pb*Lig1 cKO parasites undergo limited nuclear division and present a reduced DNA content that fails to increase beyond mid-liver stage of development. These data suggest that Lig1 is an essential enzyme for parasite blood- and liver-stage development.

**IMPORTANCE:**

Unlike mammalian cells that possess multiple DNA ligases, the malaria parasite’s nuclear genome encodes a single DNA ligase. This single DNA ligase is likely involved in both DNA replication and DNA repair. However, the importance of parasite DNA ligase remains largely unknown. Here, we show that *Plasmodium* Lig1 is primarily found within the nucleus, but it also exhibits a distribution across parasite organelles. Knockout of *Pb*Lig1 in sporozoites abolishes parasite liver-stage development, preventing the formation of hepatic merozoites and ultimately blocking the transition from the liver to the blood stage of infection. More specifically, *Pb*Lig1 is essential for nuclear division during hepatic schizogony. These findings enhance our understanding of the role of DNA ligase I in malaria parasite liver-stage development.

## INTRODUCTION

The malaria-causing parasite, *Plasmodium* spp., has three genome-containing compartments—the nucleus, mitochondrion, and apicoplast. The *Plasmodium* mitochondrial genome comprises tandem repeats of ~6 kb and is among the smallest known in nature (1). The apicoplast has a few copies of a circular genome of ~35 kb (2). Maintenance of the nuclear chromosomal DNA and the two organelle genomes is required for parasite growth and survival. Alternative end-joining, homologous recombination, and mismatch repair pathways have been proposed to be involved in the repair of *Plasmodium falciparum* nuclear DNA (3–6). Likely players in nucleotide excision repair in the nucleus have also been identified (7). Base excision repair (BER) has been reported in the parasite (8). Recent reports have suggested that the parasite mitochondrion is a major site for BER (9). DNA replication and repair mechanisms operating in the nucleus, mitochondrion, and apicoplast require a DNA ligase. The *P. falciparum* nuclear genome encodes a single functional DNA ligase (10) whose targeting to different cellular compartments remains to be established.

Ligases are widely distributed cellular proteins that play crucial roles in DNA replication, repair, and recombination. DNA ligases facilitate the formation of phosphodiester linkages at sites where there are breaks in the single strand between neighboring 3′-hydroxyl and 5′-phosphate ends in double-stranded DNA (11). DNA ligases can be categorized into two main classes on the basis of their cofactor requirements: one class utilizes NAD^+^ as a cofactor, whereas the other class relies on ATP. Enzymes encoded by eukaryotes, viruses, and archaebacteria all require ATP; eubacteria are the only organisms that possess NAD^+^-requiring DNA ligases. The ATP-dependent ligases vary in size, ranging from 30 kDa to over 100 kDa. On the other hand, NAD^+^-dependent enzymes are highly similar and consist of single protein units of 70 kDa–80 kDa (12). While ATP-dependent DNA ligases dominate in eukaryotic systems, an intriguing exception is the annotation of an NAD^+^-dependent DNA ligase in the genome of *Toxoplasma gondii* (13), a feature not observed in *Plasmodium*. This contrast may reflect differences in DNA repair capabilities between these organisms, particularly given that *Plasmodium* lacks the nonhomologous end-joining (NHEJ) pathway (5). In contrast, yeast cells encode two distinct DNA ligases: one dedicated to DNA replication and maintenance, and another involved in NHEJ-mediated repair, underscoring the functional diversification of ligases even within unicellular eukaryotes (14, 15). There is more than one DNA ligase found in mammalian cells (11); Archaea and bacteria have numerous DNA ligases (16). The DNA ligases produced by the human genes Lig1, Lig3, and Lig4 are typical representatives of the three groups of eukaryotic DNA ligases. DNA ligase I is the primary ligase involved in DNA replication and plays critical roles in DNA repair and recombination. DNA ligase III, which is found in vertebrate cells, is involved in DNA repair in the nucleus and mitochondria. DNA ligase IV is involved in repairing DNA double-strand breaks, NHEJ, and V(D)J recombination (17). It is important to note, however, that *Plasmodium* genomes contain a large fraction of genes annotated as “unknown function.” It is plausible that additional DNA ligases with unusual domains remain undiscovered *in silico*.

DNA ligases are known to be present in different forms for targeting to the nucleus and mitochondria. In *Saccharomyces cerevisiae*, the CDC9 gene responsible for encoding the *S. cerevisiae* counterpart of human Lig1 is crucial for yeast survival. An alternate translation start site produces two variations of the CDC9 DNA ligase. These variations are specifically targeted to either the nucleus or the mitochondria, and they play a crucial role in maintaining the genome in these organelles (18). The Lig3 gene, which is exclusive to vertebrates, produces three separate DNA ligase polypeptides. The DNA ligase IIIα mRNA is used to synthesize both nuclear and mitochondrial forms of DNA ligase IIIα via an alternate translation initiation mechanism, which occurs in all cells (19, 20). Furthermore, a specialized alternative splicing process occurs specifically in germ cells, where the final 3′-coding exon of DNA ligase IIIα mRNA is substituted with a distinct exon, resulting in the production of DNA ligase IIIβ mRNA (20, 21). Among plants, *Arabidopsis thaliana* Lig1 has been demonstrated to be targeted to both the nucleus and the mitochondria with the alternate use of two in-frame AUG codons, but there is no direct evidence for its targeting to chloroplasts (22).

The DNA ligase I encoded by the *P. falciparum* nuclear genome has considerable sequence and structural identity to yeast and human DNA ligase I in its catalytic core region at the C-terminus (10). In general, ATP-dependent DNA ligases have a more variable N-terminal region, and ligase I from *Plasmodium falciparum* (*Pf*LigI) differs from its human homolog in that it does not have a conserved PCNA-interacting domain at the N-terminus. Instead of the PCNA binding domain, a region with characteristics of an apicoplast targeting signal is present (10). The MitoProt prediction score for a mitochondrial targeting element in *Pf*LigI is 0.66, and its homologs in apicomplexan parasites are predicted to be part of the apicoplast and mitochondrial proteome (23–25). Recombinant *Pf*LigI has been biochemically characterized as a functional ligase with Mg^2+^ or Mn^2+^ and ATP as cofactors (10).

Considering the critical role of DNA ligases in genome maintenance, we sought to determine whether the only identifiable DNA ligase in *Plasmodium* is targeted to all three genome-containing compartments, namely the nucleus, apicoplast, and mitochondrion, in parasite cells. We show that Lig1 is expressed throughout the parasite life cycle. Using immunofluorescence and chromatin immunoprecipitation (ChIP)-PCR assays, we found that Lig1 is preferentially present in the nucleus and is also distributed to the apicoplast and mitochondria. Owing to the indispensability of the gene in the blood stage, we used the Flp/FRT-based conditional mutagenesis system to disrupt *Plasmodium berghei* Lig1 (*Pb*Lig1) in sporozoites. The absence of *Pb*LigI blocks nuclear division and parasite liver-stage development. Our data underscore the critical role of DNA ligase I in the *Plasmodium* life cycle.

## RESULTS

### Amino acid sequence analysis of DNA ligase I

**Fig 1:**
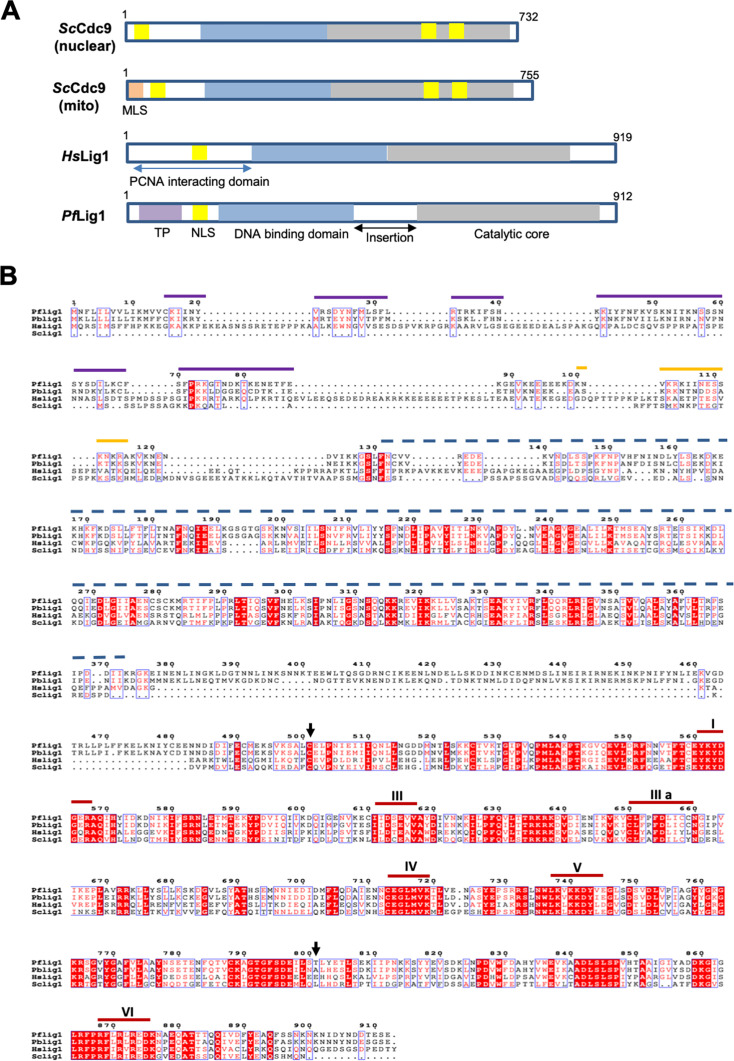
Targeting elements and domain organization (**A**) and ClustalW multiple sequence alignment (**B**) of *Pf*Lig I and its homologs in *Homo sapiens*, *Saccharomyces cerevisiae,* and *P. berghei*. The black arrows mark the first and last amino acid residues of rec*Pf*LigI; the purple lines mark the predicted transit-peptide for apicoplast targeting, the yellow line marks the NLS, and the blue dashed line indicates the DNA-binding domain of *Pf*LigI. Motifs I–VI of the catalytic domain of the protein are indicated.

*Pf*LigI and *Pb*LigI have an unconserved insertion of ~100 amino acids between the DNA binding and catalytic core domain (Fig. 1; see Fig. S1 at https://doi.org/10.6084/m9.figshare.30461318). Unique to *Pf* and *Pb*LigI is a putative apicoplast targeting sequence (10). The two *Plasmodium* proteins also have a nuclear localization signal (Fig. 1). Phylogenetic tree analysis revealed a close evolutionary relationship between organisms, indicating Lig1 likely shares a common ancestor with similar protein properties (see Fig. S2A at https://doi.org/10.6084/m9.figshare.30461318). The percent matrix similarity revealed that Lig1 is conserved within *Plasmodium* species and shows less sequence similarity with other model organisms (see Fig. S2B at https://doi.org/10.6084/m9.figshare.30461318).

### DNA ligase I is targeted to the parasite nucleus as well as organelles

**Fig 2:**
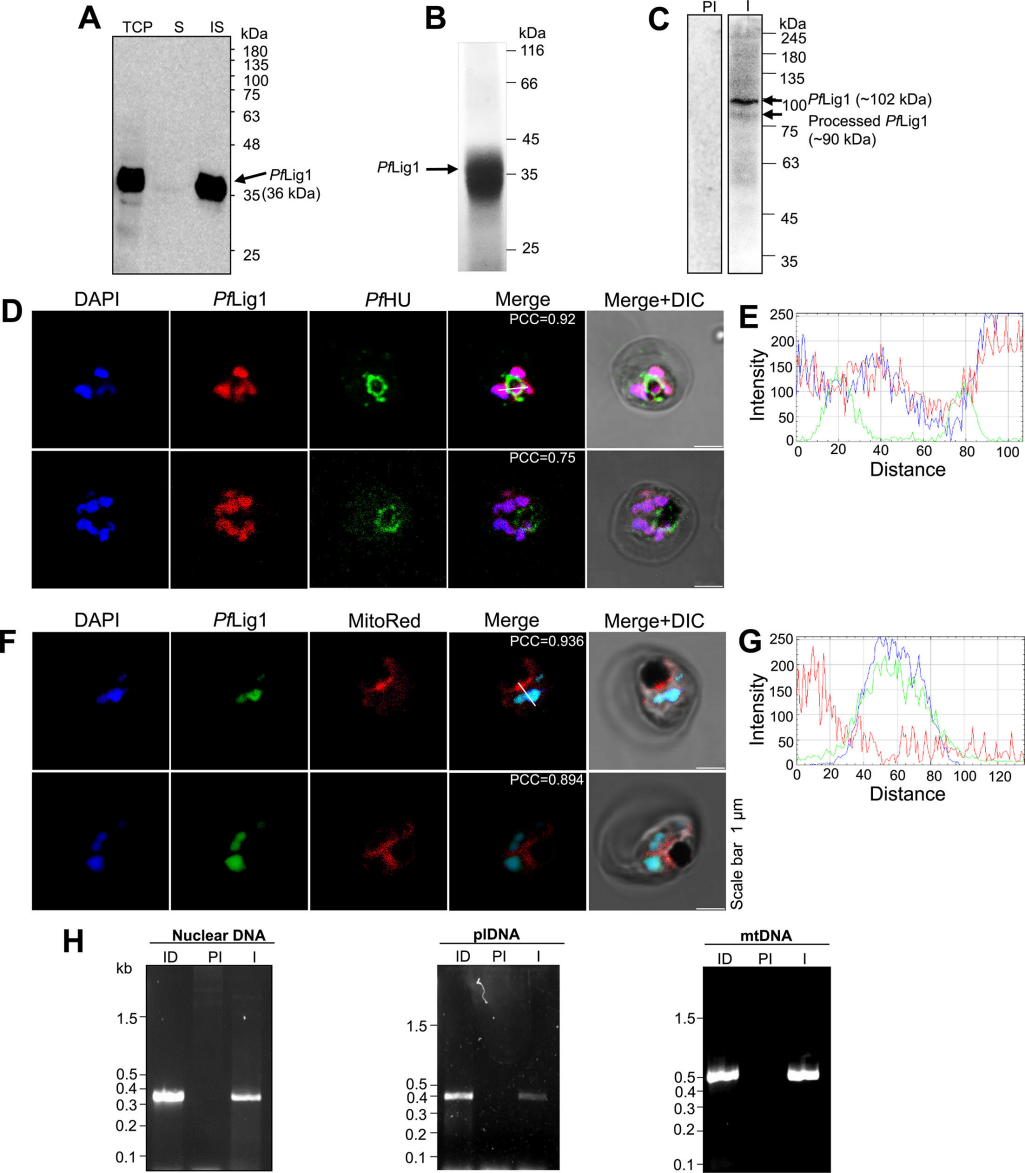
Subcellular localization and DNA association of *Pf*LigI. (**A**) Western blot analysis of recombinant *Pf*LigI catalytic domain expressed in *E. coli*; S, soluble; IS, insoluble fraction. An anti-6×His antibody detected a 36 kDa band corresponding to the 6×His-tagged *Pf*LigI catalytic domain in the insoluble fraction. (**B**) Coomassie-stained SDS-PAGE gel showing purified, electroeluted recombinant *Pf*LigI used for rabbit immunization. (**C**) Western blot analysis of *P. falciparum* parasite lysates probed with anti-*Pf*LigI immune serum identified a ~102 kDa band consistent with the predicted full-length *Pf*LigI, and a ~90 kDa band representing a possible processed form. No signal was observed with PI serum. (**D and F**) Immunofluorescence confocal microscopy of RBCs infected with parasites at the trophozoite stage probed with anti-*Pf*LigI and the apicoplast marker anti-*Pf*HU antibodies or MitoTracker Red CMXRos. DIC, differential interference contrast. To quantify colocalization, Pearson correlation coefficient (PCC) was calculated from 32 and 48 images in panels D and E, respectively. (**E**) Line profile of fluorescence intensity showing overlap of *Pf*LigI (red) and DAPI (blue), indicating colocalization with nuclear DNA. (**G**) Line profile showing overlap of *Pf*LigI (green) and DAPI (blue). (**H**) Anti-*Pf*LigI antibodies were used to pull down mitochondrial DNA (mtDNA), apicoplast DNA (plDNA), and nuclear DNA via ChIP.-PCR products were obtained using primers for a segment of the nuclear-encoded *Pf*Exo gene (0.34 kb), the apicoplast rpl16 segment (0.39 kb), and the mitochondrial fragment F (0.445 kb). ID, input DNA; I, immune serum.

To raise antibodies against *Pf*Lig1, the catalytic domain was expressed as an N-terminal 6×His-tagged fusion protein in *Escherichia coli*. Protein expression was confirmed by western blotting with an anti-6×His antibody (Fig. 2A). The protein band appeared at the expected size of ~36 kDa; however, all of the protein was present in inclusion bodies. The recombinant protein was purified from detergent-washed inclusion bodies, followed by gel electroelution after SDS-PAGE (Fig. 2B). Purified *Pf*Lig1 was used as an antigen for raising polyclonal antisera in rabbits. Anti-*Pf*LigI sera recognized a ~102 kDa band in western blots of *P. falciparum* lysate, which corresponds to the expected size of the full-length protein, and a lower processed band at ~90 kDa corresponding to the size expected for a signal- and transit-peptide-cleaved protein for apicoplast targeting (Fig. 1 and 2C). The processed band was of lower intensity than the full-length protein. The control pre-immune (PI) serum did not recognize any protein in the lysate. To investigate the subcellular localization of *Pf*Lig1, blood-stage parasites were immunostained with anti-*Pf*Lig1 or anti-*Pf*HU antibodies (apicoplast marker) or MitoTracker Red CMXRos (mitochondrial dye). Nuclei were stained with DAPI. There was a prominent *Pf*Lig1 signal in the nuclei (Fig. 2D through G). However, extranuclear signals of *Pf*Lig1 were not observed, and no overlap of *Pf*Lig1 with apicoplast (Fig. 2D and E) or mitochondrial (Fig. 2F and G) markers was detected. We thus considered the possibility that the prominent fluorescence signal in the nucleus masks the possible low levels of *Pf*Lig1 in the organelles. To address this, a ChIP-PCR assay was carried out using anti-*Pf*Lig1 antibodies. Anti-*Pf*LigI antibodies pulled down all three (nuclear, mitochondrial, and apicoplast) genomes, whereas no signal was detected in ChIP-PCRs after pull-down with pre-immune serum (Fig. 2H). Detection of the interaction of the protein with the apicoplast and mitochondrial genomes suggests that *Pf*LigI may be present in both organelles and could interact with their genomes.

**Fig 3:**
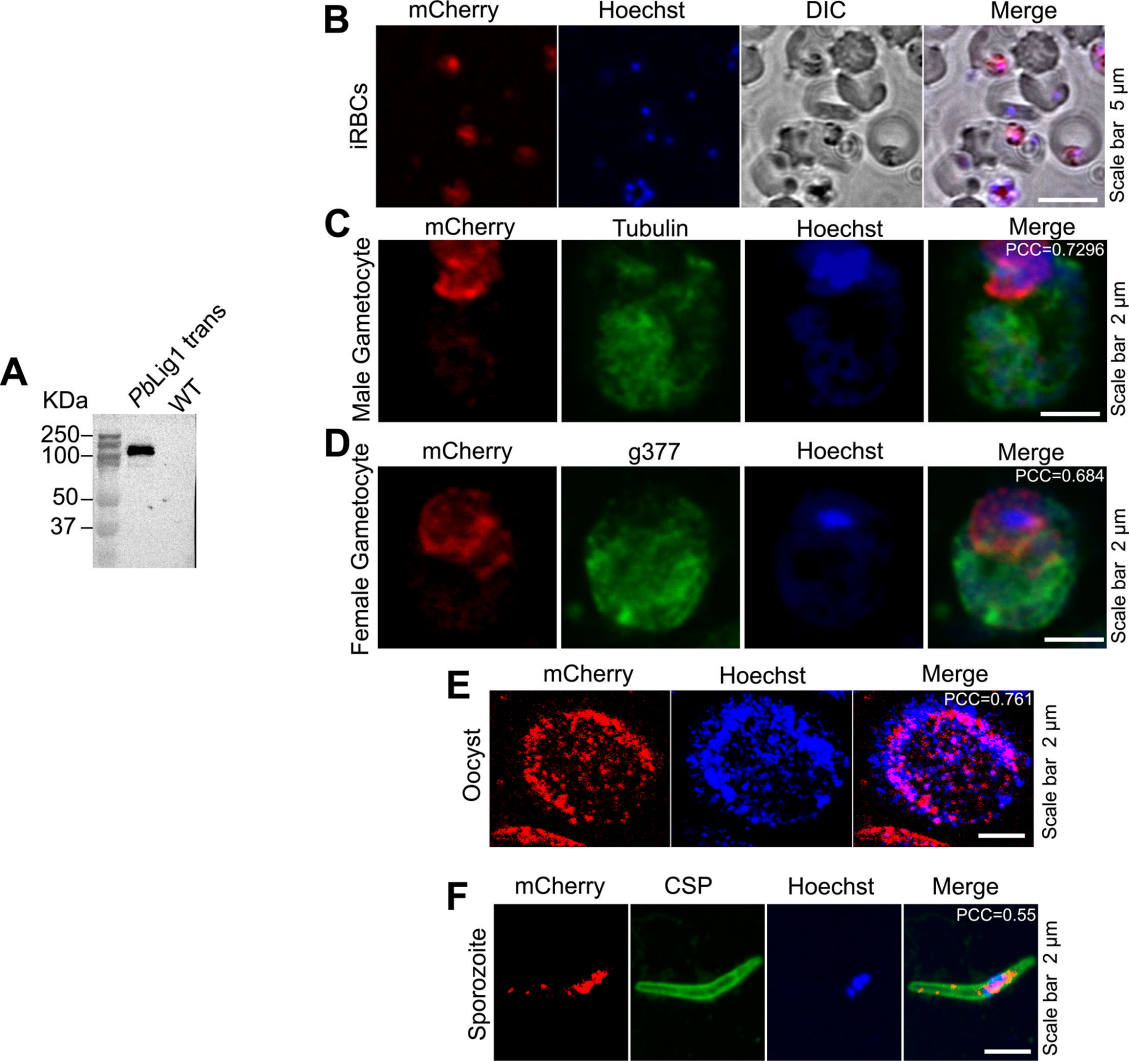
Expression and localization of Lig1 in *P. berghei*. (**A**) Western blot analysis of blood-stage parasites expressing *Pb*Lig1-3×HA-mCherry. Lysates from transgenic and WT parasites were probed with an anti-mCherry antibody. A specific band corresponding to the *Pb*Lig1-3×HA-mCherry fusion protein was detected in the transgenic line, but not in WT, confirming successful expression. (**B–D**) Immunofluorescence analysis of *Pb*Lig1-3×HA-mCherry in iRBCs and gametocytes. An anti-mCherry antibody was used to detect the fusion protein. Stage-specific markers were used for co-staining: anti-HSP70 for asexual blood stages, anti-α-tubulin for male gametocytes, and anti-G377 for female gametocytes. *Pb*Lig1-3×HA-mCherry was detected in both asexual and sexual blood-stage parasites, with distinct localization patterns. (**E**) mCherry fluorescence of the *Pb*Lig1-3×HA-mCherry parasite in the oocyst in relation to the nucleus. (**F**) Expression of *Pb*Lig1-3×HA-mCherry in salivary gland sporozoites. Sporozoites were identified using an anti-CSP antibody. PCC was calculated from 15 images for panels C, D, and E, and from 10 images for panel F.

**Fig 4:**
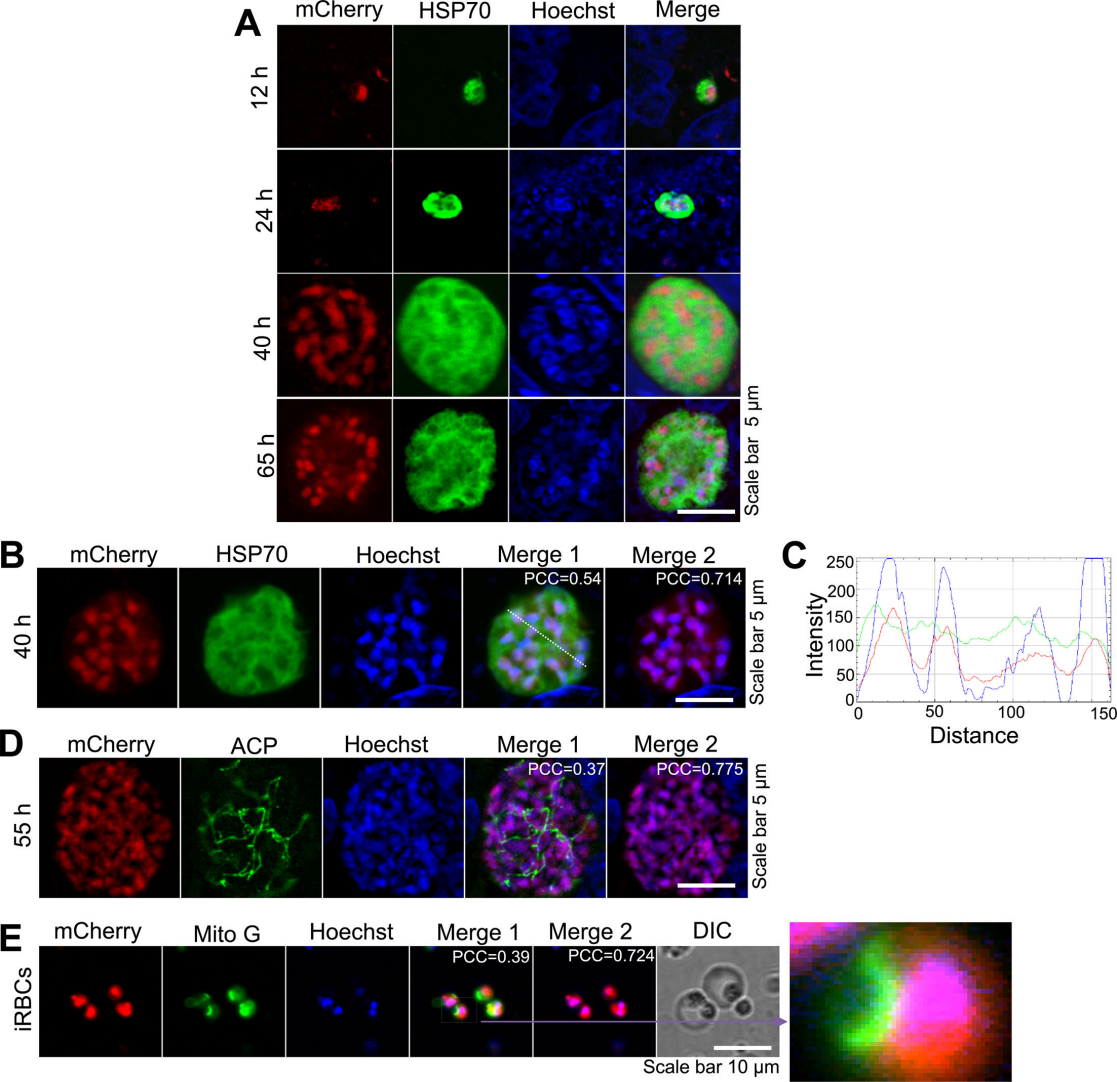
Localization of Lig1 in *P. berghei*. (**A**) Fluorescence microscopy images showing expression of *Pb*Lig1-3×HA-mCherry in EEFs at 12, 24, 40, and 65 hpi. mCherry fluorescence is localized with nuclei, which were stained with Hoechst 33342. (**B**) *Pb*Lig1-3×HA-mCherry fluorescence colocalizes with Hoechst-stained nuclei, indicating nuclear-specific localization. (**C**) Fluorescence intensity profiles of mCherry (red) and Hoechst (blue) along the dotted line reveal overlapping peaks, indicating colocalization of Lig1 with nuclear DNA. (**D**) *Pb*Lig1-3×HA-mCherry fluorescence signal does not overlap with the apicoplast marker ACP. (**E**) *Pb*Lig1-3×HA-mCherry signal does not colocalize with the mitochondrial marker in iRBCs. Nuclei were stained with Hoechst 33342. PCC was calculated from 20 images for panel B, and from 10 images for panels C and D.

To investigate the expression and localization of Lig1 in the rodent malaria parasite *P. berghei*, we generated a transgenic line expressing a C-terminally tagged *Pb*Lig1-3×HA-mCherry fusion protein (see Fig. S3A at https://doi.org/10.6084/m9.figshare.30461318). Correct integration and expression were confirmed by diagnostic PCR and western blotting (Fig. 3A; see Fig. S3B at https://doi.org/10.6084/m9.figshare.30461318). An anti-mCherry antibody detected a protein band of the expected size (~127.5 kDa) in transgenic parasites but not in wild-type (WT) controls (Fig. 3A). Live imaging of infected red blood cells (iRBCs) confirmed mCherry fluorescence (Fig. 3B). We next examined the subcellular localization of *Pb*Lig1 during various life cycle stages using immunofluorescence assays (IFA) with antibodies against cytosolic HSP70 and the apicoplast marker ACP. *Pb*Lig1 was expressed throughout the parasite life cycle, including asexual blood stages, male and female gametocytes, oocysts, sporozoites, and EEFs, and localized predominantly to the nucleus (Fig. 3B through F and 4A; see Fig. S3C at https://doi.org/10.6084/m9.figshare.30461318). No detectable *Pb*Lig1 signal was observed in mitochondria or apicoplasts, possibly due to the dominant nuclear localization masking weaker signals in other organelles (Fig. 4B through E), consistent with observations for *P. falciparum* Lig1.

### *Pb*Lig1 is essential for the development of *P. berghei* blood stages

**Fig 5:**
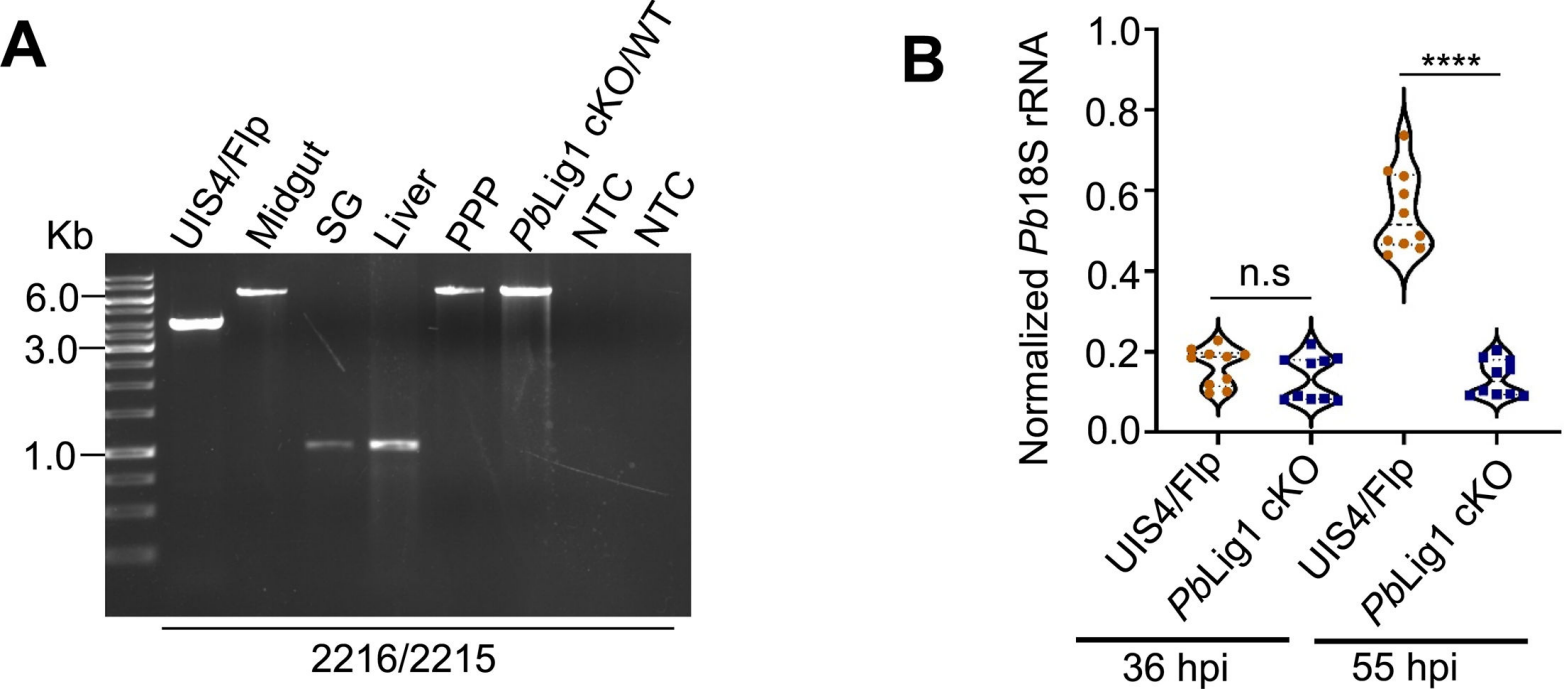
*Pb*Lig1 is essential for infection in mice. (**A**) To assess excision of the *Pb*Lig1 locus, genomic DNA was isolated from *Pb*Lig1 cKO parasites at various life cycle stages, including sporozoites, infected hepatocytes, and iRBC. Diagnostic PCR using primers 2216 and 2215 detected the excised locus (1.1 kb band) in sporozoites and liver-stage parasites, indicating successful excision in these stages. In contrast, parasites that established patent blood-stage infections in mice retained the nonexcised locus. Parasites transfected with the *Pb*Lig1 cKO construct but not induced for excision showed only the nonexcised band. NTC, no-template control. (**B**) To evaluate liver-stage parasite development, *Pb*Lig1 cKO and UIS4/Flp sporozoites were injected intravenously into C57BL/6 mice. Livers were harvested at 36 and 55 hpi, and parasite burden was quantified by qPCR by measuring *Pb*18S rRNA, normalized to mouse GAPDH. At 36 hpi, parasite loads were comparable between groups (*P* = 0.6661), n.s., not significant. By 55 hpi, *Pb*Lig1 cKO parasites showed a significant reduction in liver burden compared to controls (****P < 0.0001), indicating a block in late liver-stage development. Data represent the mean ± SD from two independent biological replicates (*n* = 5 mice per group).

Previous attempts to disrupt Lig1 in *P. berghei* (26) or *P. falciparum* (27) were unsuccessful in high-throughput genetic screening. We obtained *Pb*Lig1 targeting plasmid from PlasmoGEM and attempted to delete the gene but failed (see Fig. S4A at https://doi.org/10.6084/m9.figshare.30461318). Therefore, we used the Flp/FRT-based conditional mutagenesis system, which preserves gene function during the blood-to-mosquito stages and silences it only once the sporozoites reach the salivary glands. We first generated a targeting construct, pLig1/FRT-intron/TRAP3′UTR, designed to insert an FRT site within the intron and replace the native 3′UTR with that of TRAP (see Fig. S4B at https://doi.org/10.6084/m9.figshare.30461318). Transfection of this construct into *P. berghei* UIS4/Flp schizonts failed to yield drug-resistant parasites after three independent attempts, suggesting that insertion of the FRT site within the intron or replacement of the 3′UTR disrupts *Pb*Lig1 expression and is lethal. To distinguish between these possibilities, we generated pLig1/FRT-TRAP3′UTR, a construct designed to replace only the 3′UTR of *Pb*Lig1 with that of TRAP, and again failed to recover drug-resistant parasites following transfection (see Fig. S4C at https://doi.org/10.6084/m9.figshare.30461318). These results indicate that the TRAP 3′UTR does not support *Pb*Lig1 expression, and the native 3′UTR is required for gene function. We next generated the construct pLig1/FRT-intron, designed to insert an FRT site within the intron without altering the 3′UTR (see Fig. S5A at https://doi.org/10.6084/m9.figshare.30461318). Transfection into UIS4/Flp parasites yielded drug-resistant parasites, and correct site-specific integration was confirmed by diagnostic PCR (see Fig. S5B at https://doi.org/10.6084/m9.figshare.30461318) and XbaI digestion (see Fig. S5C and D at https://doi.org/10.6084/m9.figshare.30461318). This construct was also transfected into *P. berghei* WT parasites to generate a control line (see Fig. S5E at https://doi.org/10.6084/m9.figshare.30461318), ensuring that any observed phenotype upon excision is specific to *Pb*Lig1 deletion. To assess whether the genetic modifications affected blood-stage development, Swiss albino mice were intravenously (i.v.) injected with equal numbers of iRBCs, and parasitemia was monitored by Giemsa-stained blood smears. Asexual blood-stage growth of *Pb*Lig1 cKO parasites was comparable to that of the UIS4/Flp control (see Fig. S5F at https://doi.org/10.6084/m9.figshare.30461318). Similarly, mosquito-stage development was not affected; oocyst numbers, as well as midgut and salivary gland sporozoite counts, were comparable among *Pb*Lig1 cKO, UIS4/Flp, and *Pb*Lig1 cKO/WT control parasites (see Fig. S6A through E at https://doi.org/10.6084/m9.figshare.30461318). To confirm excision of the FRT-flanked *Pb*Lig1 locus in sporozoites, genotyping was performed on parasites isolated from salivary glands. Efficient excision was observed in the UIS4/Flp line, which expresses Flp recombinase during sporozoite stages, but not in WT parasites lacking Flp expression (Fig. 5A). These data demonstrate successful stage-specific deletion of *Pb*Lig1 and establish that the gene is essential for asexual blood-stage development. Moreover, disruption of *Pb*Lig1 expression by alteration of its 3′UTR or gene excision abrogates parasite viability, indicating that both gene integrity and regulated expression are critical for function.

### Lig1 is essential for *P. berghei* liver-stage development

**Fig 6:**
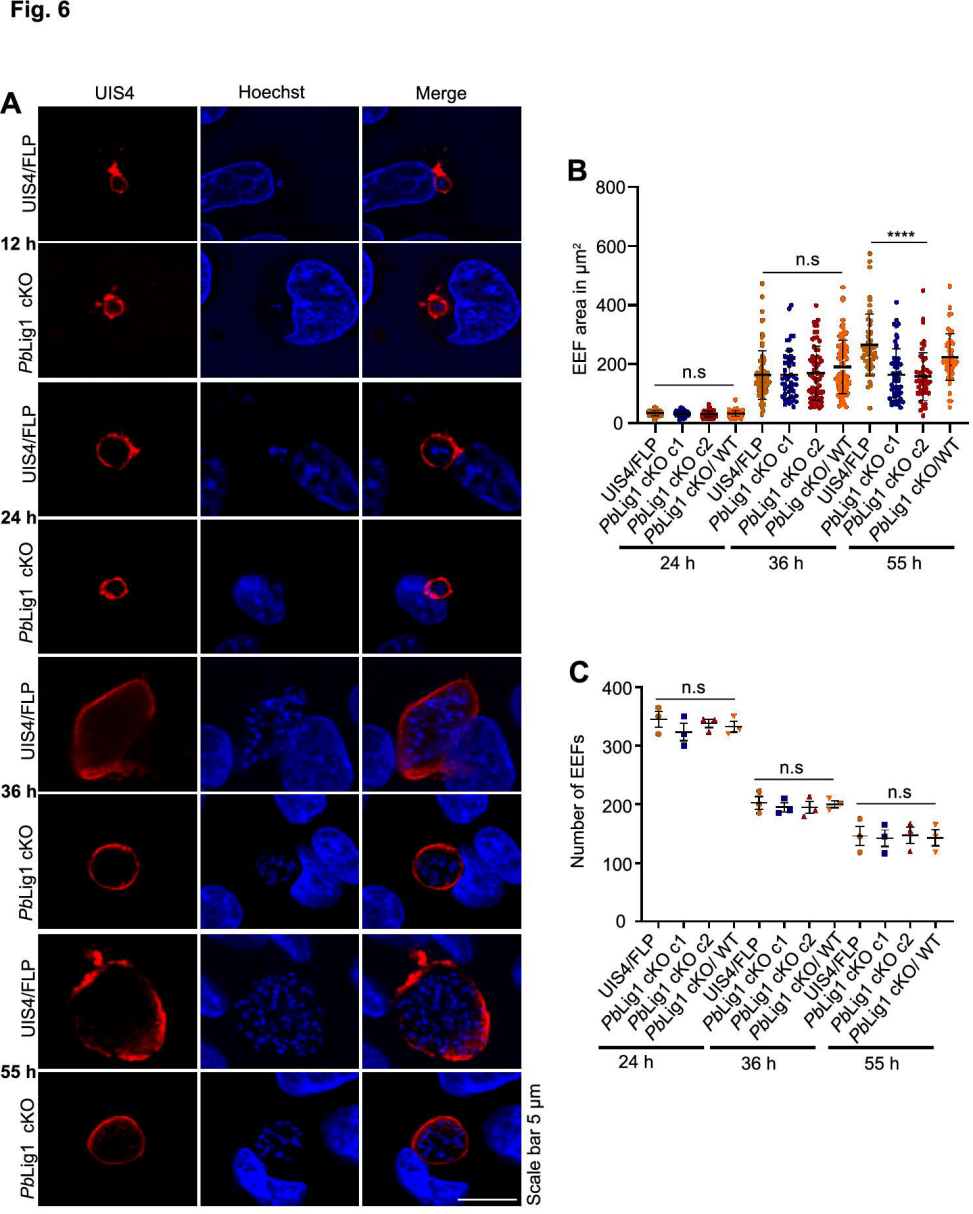
*Pb*Lig1 is essential for parasite development in the liver. (**A**) HepG2 cells were infected with either UIS4/Flp or *Pb*Lig1 cKO sporozoites. Infected cultures were fixed at the indicated time points post-infection and immunostained with an anti-UIS4 antibody to visualize EEFs. (**B**) EEF area was comparable at 24 hpi (*P* = 0.2500) and 36 hpi (*P* = 0.1840), but was significantly reduced in *Pb*Lig1 cKO parasites at 55 hpi (****P < 0.0001). Data represent the mean ± SEM from three independent experiments. (**C**) Quantification of EEF numbers at 24, 36, and 55 hpi revealed no significant differences between UIS4/Flp and *Pb*Lig1 cKO parasites (24 hpi, *P* = 0.6063; 36 hpi, *P* = 0.9036; 55 hpi, *P* = 0.9929; one-way ANOVA).

To evaluate the role of *Pb*Lig1 in mammalian host infectivity, C57BL/6 mice were intravenously inoculated with salivary gland sporozoites from the *Pb*Lig1 cKO line. All mice infected with UIS4/Flp parasites developed blood-stage parasitemia by day 3 post-infection (p.i.), whereas mice inoculated with *Pb*Lig1 cKO sporozoites failed to establish blood-stage infection (Table 1). Notably, the few mice that became patent harbored parasites retaining an intact *Pb*Lig1 locus, indicating incomplete gene excision (Fig. 5A). To quantify liver-stage development, parasite burden was assessed by measuring *Pb*18S rRNA transcript levels. At 36 hpi, *Pb*Lig1 cKO parasites exhibited liver-stage burdens comparable to those of UIS4/Flp. However, transcript levels were significantly reduced in *Pb*Lig1 cKO-infected livers by 55 hpi (Fig. 5B), indicating a developmental arrest. To further investigate this phenotype, HepG2 cells were infected with *Pb*Lig1 cKO sporozoites and fixed at 12, 24, 36, and 55 hpi for immunofluorescence analysis using anti-UIS4 antibodies (Fig. 6A). *Pb*Lig1 cKO and UIS4/Flp parasites showed comparable growth through 36 hpi; however, *Pb*Lig1 cKO EEFs failed to increase in size beyond this point (Fig. 6B). Importantly, the number of EEFs was similar between groups at all time points (Fig. 6C). Together, these data demonstrate that *Pb*Lig1 is dispensable for early liver-stage development but is essential for mid- to late-liver-stage maturation and successful transition to the blood stage.

**TABLE 1:** Infectivity of *Pb*Lig1 cKO sporozoites in C57BL/6 mice^*a*^

Exp.	Parasites	Number of sporozoites injected	Mice positive/mice injected	Prepatent period (days)
1	UIS4/Flp	5,000	5/5	3
	*Pb*Lig1 cKO c1	5,000	0/5	NA^*b*^
	*Pb*Lig1 cKO c2	5,000	1/5	9
	*Pb*Lig1 cKO/WT	5,000	5/5	3.4
2	UIS4/Flp	5,000	5/5	3.2
	*Pb*Lig1 cKO c1	5,000	1/5	8
	*Pb*Lig1 cKO c2	5,000	0/5	NA
3	UIS4/Flp	5,000	5/5	3.2
	*Pb*Lig1 cKO c1	5,000	0/5	NA

^*a*^ Groups of mice were infected intravenously with salivary gland sporozoites.

^*b*^ NA, not applicable.

### *Pb*Lig1 cKO EEFs exhibit defective nuclear division and reduced DNA content

**Fig 7:**
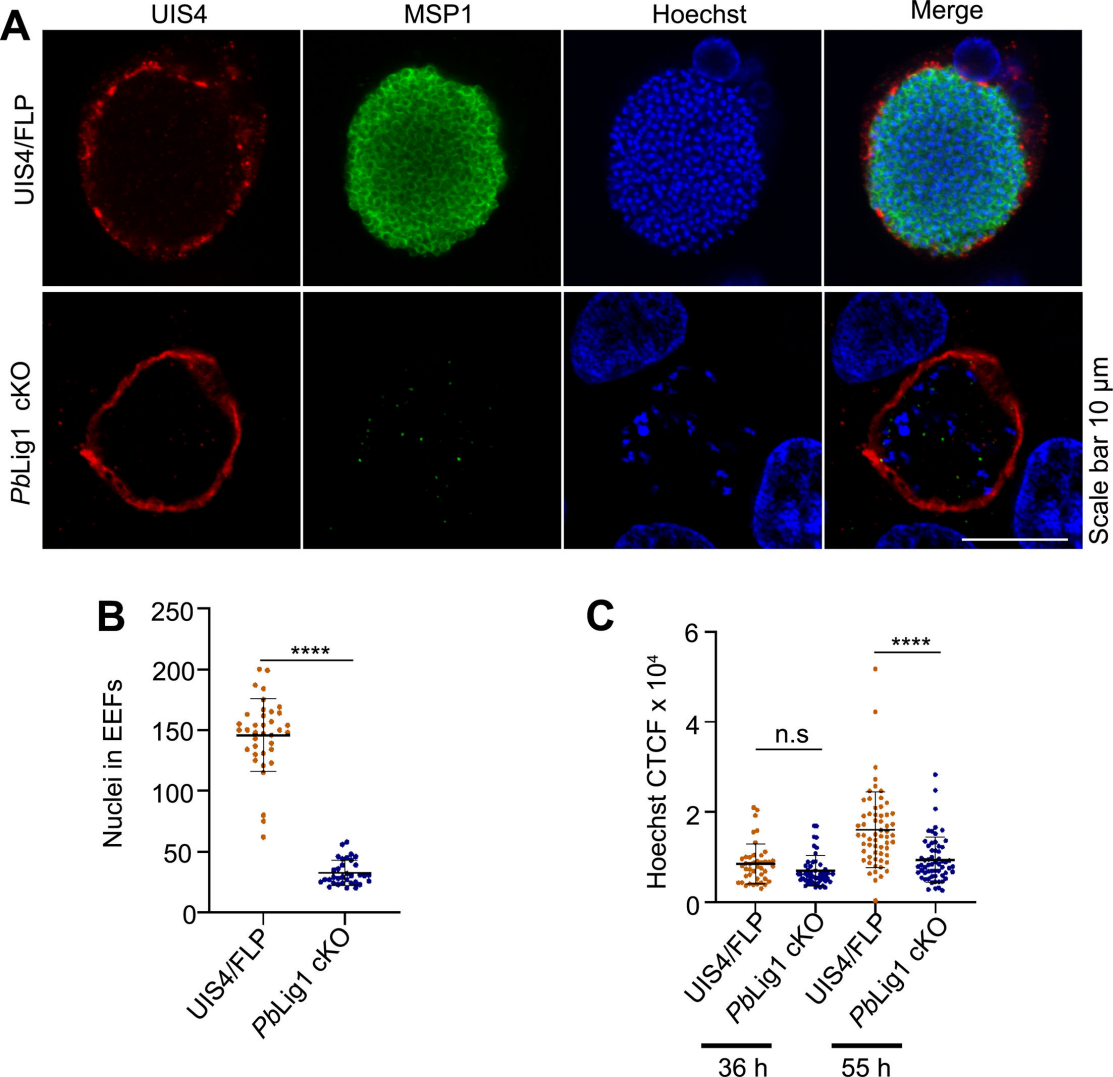
*Pb*Lig1 is essential for nuclear division and merozoite development. (**A**) HepG2 cells infected with UIS4/Flp or *Pb*Lig1 cKO sporozoites were fixed at 62 hpi and immunostained with anti-UIS4 and anti-MSP1 antibodies. UIS4/Flp parasites exhibited robust MSP1 staining, indicative of merozoite formation, whereas *Pb*Lig1 cKO parasites lacked MSP1 signal, suggesting a block in merozoite development. (**B**) Quantification of nuclear number at 55 hpi. Nuclei were manually counted in ImageJ-analyzed images. *Pb*Lig1 cKO EEFs displayed a significantly reduced number of nuclei compared to UIS4/Flp (****P < 0.0001, unpaired *t*-test). (**C**) Quantification of DNA content using Hoechst 33342 staining at 36 hpi and 55 hpi. UIS4/Flp and *Pb*Lig1 cKO EEFs were fixed and immunostained with anti-UIS4 and anti-MSP1 antibodies. Nuclear DNA within the PV was quantified by calculating corrected total cell fluorescence (CTCF) from Hoechst signal. Data represent 43 (UIS4/Flp 36 hpi), 47 (*Pb*Lig1 cKO 36 hpi), 60 (UIS4/Flp 55 hpi), and 60 (*Pb*Lig1 cKO 55 hpi) individual EEFs. No significant difference in CTCF was observed at 36 hpi (*P* = 0.0693), while a significant reduction was detected in *Pb*Lig1 cKO parasites at 55 hpi (****P < 0.0001, unpaired *t*-test), n.s., not significant. Data are shown as mean ± SD from two independent biological replicates.

To evaluate merozoite development, *Pb*Lig1 cKO EEFs harvested at 62 hpi were immunostained with anti-MSP1 antibodies. Merozoite formation was readily detected in UIS4/Flp cultures but was absent in *Pb*Lig1 cKO cultures (Fig. 7A). Consistently, *Pb*Lig1 cKO parasites showed a significant reduction in nuclear number compared to UIS4/Flp (Fig. 7B). Quantification of Hoechst fluorescence revealed comparable nuclear content between the two groups at 36 hpi; however, by 55 hpi, *Pb*Lig1 cKO parasites exhibited a marked decrease in DNA content relative to UIS4/Flp parasites (Fig. 7C). These results demonstrate that *Pb*Lig1 is critical for nuclear division and merozoite formation during liver-stage development.

## DISCUSSION

Malaria parasites primarily replicate asexually as haploid cells within their mammalian hosts, specifically in the liver and red blood cells. During this process, they are vulnerable to DNA damage from both the host immune response and chemotherapeutic agents. To survive and propagate, the parasite has evolved mechanisms to cope with these damaging agents. In DNA repair pathways, multiple mechanisms converge at the final step of nick sealing, where DNA ligases play crucial roles. In higher eukaryotes, these ligases are often specialized, with some dedicated to DNA repair and others involved in DNA replication (28). *Plasmodium* contains a single DNA ligase that ensures efficient DNA replication and repair (10). Our immunofluorescence assays detected *Pf*Lig1 predominantly in the nucleus, potentially reflecting low protein abundance in the mitochondrion and apicoplast. However, ChIP revealed *Pf*Lig1 association with all three genome-containing organelles, the nucleus, mitochondrion, and apicoplast, indicating its functional involvement in maintaining genome stability across all compartments. These results suggest that *Pf*Lig1 is a central component of the parasite’s DNA replication and repair machinery in all organellar genomes. Discrepancies between IFA and ChIP results likely reflect differences in sensitivity and detection thresholds. Similar observations have been reported for other proteins; for example, MCP1 was localized to the cytoplasm by IFA (29), whereas ChIP analysis indicated exclusive nuclear association (30).

The functional interplay between DNA ligase I and other proteins involved in DNA replication and repair is essential for understanding its *in vivo* activity. In humans, DNA ligase I interacts with PCNA, and this interaction is known to stimulate ligase activity (31). Although the N-terminal region of *Pf*LigI lacks the canonical proline-rich PCNA-interacting motif found in human LigI, it may harbor a distinct PCNA-binding domain unique to Apicomplexan parasites (10). Among the three human DNA ligases, LigI serves as the primary replicative enzyme, playing a critical role in the maturation of Okazaki fragments during lagging-strand synthesis. Importantly, LigI also exhibits substrate specificity, ligating RNA-DNA junctions only when RNA is positioned 5′ to the nick, while discriminating against junctions where RNA is 3′ to the nick (32, 33). *Pf*LigI mirrors this behavior, suggesting functional conservation in substrate recognition (10). Beyond its role in DNA replication, *Pf*LigI likely participates in nuclear and organellar DNA repair. Both BER and double-strand break repair pathways have been reported in the *Plasmodium* mitochondrion (9, 34), and *Pf*LigI represents the sole identified ligase capable of maintaining organellar genome integrity.

To investigate the role of *Pb*Lig1, we attempted to delete the gene but were unsuccessful, consistent with previous reports in *P. berghei* (26) and *P. falciparum* (27). The inability to disrupt *Pb*Lig1 suggests it is essential during the asexual blood stage. To explore its function during liver-stage development, we employed the Flp/FRT recombination system (35) to conditionally delete *Pb*Lig1 in sporozoites. Conditional knockout parasites progressed normally through early liver-stage development, forming EEFs, indicating that *Pb*Lig1 is not required for initial liver stage establishment. However, *Pb*Lig1-deficient parasites exhibited a marked developmental arrest during mid-liver stage, failing to complete replication or produce hepatic merozoites in both *in vitro* and *in vivo* settings. This phenotype closely resembles the developmental arrest seen in radiation-attenuated sporozoites (36). Early abortion of liver-stage development, infection with RAS, has been shown, indicating that uninucleate trophozoites may not have been able to start the process of cell division (37). In contrast, *Pb*Lig1 cKO parasites were able to enter nuclear division but failed to complete replication, suggesting a defect during the growth and replication phase of liver-stage development. We hypothesize that this arrest results from impaired ligation of Okazaki fragments due to the absence of *Pb*Lig1, leading to DNA replication stress or genomic instability (38). Notably, this phenotype differs from that of parasites lacking BER enzymes, which complete liver-stage development but fail to initiate blood-stage infection (9, 34).

Okazaki fragments continue to be synthesized on the lagging strand even after leading strand synthesis has stopped. In most eukaryotic systems, cells mitigate the accumulation of unligated Okazaki fragments through an alternative ligation pathway (38, 39). Although DNA ligase I is the primary enzyme responsible for sealing nicks between Okazaki fragments, recent studies suggest that Lig3, in complex with XRCC1, can compensate for Lig1 dysfunction in certain contexts (39). In contrast, the *Plasmodium* genome encodes only a single DNA ligase, suggesting a lack of redundancy in Okazaki fragment processing. This dependency may render the parasite particularly susceptible to replication stress when Lig1 is compromised. In *Pb*Lig1 cKO EEFs, the fate of unligated Okazaki fragments remains unclear; however, their accumulation may contribute to replication fork stalling and collapse.

In conclusion, we show that DNA ligase I localizes primarily to the nucleus but is also detected in the apicoplast and mitochondria. Collectively, our findings demonstrate that Lig1 is essential for nuclear division during liver-stage development of *Plasmodium*. Further investigation is needed to define the specific roles of Lig1 in nuclear and organellar DNA replication and repair in malaria parasites.

## MATERIALS AND METHODS

### Parasites

*P. falciparum* 3D7 and *P. berghei* ANKA (MRA 311) parasite lines were obtained from BEI Resources, USA. *P. berghei* ANKA expressing Flp (flippase) under the stage-specific promoter UIS4 was used for the generation of conditional knockout parasites (S. Mishra, K.A. Kumar, and P. Sinnis, unpublished data). *P. falciparum* was cultured in human erythrocytes maintained in complete RPMI-1640 medium (RPMI-1640 with L-glutamine and 25 mM HEPES supplemented with 1% glucose, 0.2% NaHCO3, and 0.5% Albumax II). Hypoxanthine (13.6 µg/mL) and gentamicin sulfate (25 µg/mL) were added to the culture medium.

### Animals, mosquitoes, and cell lines

For routine *P. berghei* infection and to maintain the mosquito breeding cycle, female Swiss albino mice (6–8 weeks old) were used. Female C57BL/6 mice (6–8 weeks old) were used for sporozoite *in vivo* infection. A New Zealand white rabbit was used for immunization and the generation of antiserum. Mosquitoes were reared and maintained at 28°C and 80% relative humidity with a 12 h light/dark cycle at the CSIR-Central Drug Research Institute insectary facility. Mosquito stages of parasites were obtained by infecting female *Anopheles stephensi* and were kept at 19°C and 80% relative humidity, as described previously (40). Human liver hepatocellular carcinoma (HepG2) cells (ATCC) were used for *in vitro* infection of sporozoites.

### Bioinformatic analysis

We retrieved protein FASTA sequences from PlasmoDB and then utilized NCBI BLASTp to search for similar sequences in protein databases. The sequences were aligned using the ClustalW algorithm within the Molecular Evolutionary Genetics Analysis (MEGA12) software (41). Then, a phylogenetic tree was constructed using the maximum-likelihood method, also within MEGA12. A sequence similarity matrix was generated via UniProtAlign (42).

### Recombinant protein expression

Gene-specific primers were designed to amplify the DNA sequence encoding a segment of *Pf*Lig1 (amino acids 501–800), which was cloned into the pQE30 expression vector (containing a 6×His tag at the N-terminus) at the BamHI and SalI sites to generate pQE30-*Pf*Lig1. The clone was confirmed by restriction digestion and DNA sequencing. The recombinant *Pf*Lig1 was expressed in the *E. coli* BL21(C43) strain cotransformed with pQE30-*Pf*Lig1 and the RIG plasmid. Bacteria were grown in LB media supplemented with ampicillin (100 µg/mL) and chloramphenicol (25 µg/mL) until OD_600_ reached 0.6, followed by induction with 0.5 mM IPTG at 20°C for 16 h. The cells were harvested by pelleting at 3,663 × *g* for 10 min at 4°C and resuspended in lysis buffer (50 mM Tris-HCl, pH 7.5, 300 mM NaCl, 10% glycerol, and 1 mM PMSF). After sonication at 28% amplitude for 45 min with 20 s on/off pulses, the homogenates were centrifuged at 10,174 × *g* for 45 min at 4°C. The recombinant protein band appeared in the insoluble fraction with inclusion bodies.

### Generation of antisera and detection of Lig1 in the parasite

To generate antibodies against *Pf*Lig1, the protein was purified after washing inclusion bodies with 0.05% Triton X-100 in lysis buffer, followed by SDS-PAGE and gel electroelution in 10 mM EDTA. Antisera were generated by subcutaneous immunization in a rabbit. PI serum was collected from the rabbit before administering the antigen. Protein (200 µg) in Freund’s complete adjuvant (1:1 vol/vol) was injected as a primary immunization, followed by two booster doses (100 µg) in Freund’s incomplete adjuvant. The interval between the two booster doses was about 2 weeks (10–14 days). Ten days after the second booster, the animal was bled to obtain polyclonal antiserum. Blood was stored at 37°C for 30 min and then incubated overnight at 4°C for the separation of the serum. For detection of protein expression, parasites in mid- to late-trophozoite stage were harvested by 0.05% saponin lysis, washed twice with 1× PBS, resuspended in RIPA buffer (50 mM Tris-HCl, pH 7.4, 300 mM NaCl, 1% Triton X-100, 0.5% sodium deoxycholate, 0.1% SDS) with 1× protease inhibitor cocktail (Sigma) and incubated for 15 min, followed by sonication and centrifugation at 14,650 × *g* for 10 min at 4°C. After electrophoresis of the supernatant via SDS-PAGE, western blotting of the total cell lysate was carried out using different dilutions of antiserum as the primary Ab and goat anti-rabbit HRP (Sigma‒Aldrich) as the secondary Ab. Signals were detected using a chemiluminescence detection system (Millipore).

### Immunofluorescence staining of *P. falciparum*-infected RBCs

*P. falciparum*-infected RBCs at the mid‒late trophozoite stage and parasitemia of 10%–15% were processed for immunofluorescence labeling and confocal microscopy as described previously (43). Cells were fixed in PBS containing 4% paraformaldehyde (PFA) and 0.0075% (vol/vol) glutaraldehyde and incubated with the fixing agent on a tube rotator for 30 min. Fixed cells were washed with PBS before permeabilization with 0.1% (vol/vol) Triton X-100 in PBS for 20 min at room temperature on a tube rotator. After four washes with PBS, cells were blocked in 3% BSA in PBS for 1 h at 4°C and incubated overnight in anti-*Pf*Lig1 antisera (1:50). Anti-HUp Ab (1:200) (43) was used as a marker for the apicoplast. After washing five times with PBS, cells were probed with Alexa Fluor 488-tagged anti-mouse Ab (Invitrogen) (to detect *Pf*HU) and Alexa Fluor 568-tagged anti-rabbit Ab (Invitrogen) (to detect *Pf*Lig1) (1:1,000) in 3% BSA (in PBS). DAPI (20 µg/mL) prepared in PBS was added to the secondary Ab mixture for nuclear staining. Cells in the secondary Ab mixture were incubated on poly-L-lysine-coated glass coverslips for 2 h at room temperature. For mitochondrial staining, cells were incubated with 50 nM MitoTracker Red CMXROS (Invitrogen) for 30 min at 37°C before fixing; anti-rabbit Alexa Fluor 514-tagged Ab (Invitrogen) was used as secondary Ab for *Pf*Lig1 in MitoTracker-stained cells. After incubation, coverslips were gently washed five times with 500 µL cold PBS to remove unadhered cells. Coverslips were mounted on anti-fade mounting media, and imaging was carried out on a Leica SP8 confocal microscope using a 63× oil-immersion objective.

### ChIP-PCR

*P. falciparum* culture, primarily at the late trophozoite stage, was processed to perform ChIP (9). *P. falciparum* culture was treated with 1% (vol/vol) formaldehyde at 37°C for 15 min to induce protein-DNA cross-linking. Parasites were harvested via 0.05% (wt/vol) saponin lysis. The parasite pellet was washed with 1× PBS, and the cells were then resuspended in 1 mL ChIP buffer (30 mM Tris-HCl, pH 8.0, 150 mM NaCl, 1 mM EDTA, 0.5% Triton X-100, and 1% NP-40) and incubated on ice for 20 min. Resuspended cells were then sonicated nine times with pulses of 30% amplitude for 10 s each and cooled for 1 min between sonication cycles. The supernatant obtained after centrifugation at 14,000 × *g* for 10 min at 4°C, carrying soluble chromatin, was precleared with 50 µL of 50% Protein A Sepharose CL-4B (GE Healthcare/Cytiva) and 20 µg of sheared salmon-sperm DNA (Sigma) for 2 h at 4°C. The beads were then centrifuged at 10,000 × *g* for 2 min. Fifty microliters of the supernatant was set aside as a control input DNA. Pre-immune serum or anti-*Pf*Lig1 serum (1:100) was added to the remaining supernatant, which was subsequently incubated at 4°C for 2 h. A 50% slurry of Protein A Sepharose (50 µL) and salmon-sperm DNA (20 µg) was added to the mixture and incubated overnight on a tube rotator at 4°C. After centrifugation, the Sepharose beads were washed three times with ChIP buffer. The beads were washed twice with 1× TE (10 mM Tris, pH 8, 1 mM EDTA), and once with 1× TE containing 0.01% SDS. 1× TE supplemented with 1% SDS was used to elute the bound chromatin. Reverse cross-linking of eluted chromatin was performed by heating at 65°C for 6 h. Chromatin was then treated with proteinase K (20 µg) for 2 h at 42°C. DNA was extracted with phenol twice and then with phenol:chloroform (1:1). DNA was precipitated with ethanol, followed by washing with 70% ethanol. DNA from the input and ChIP samples was used as a template for PCR amplification. Primers for the nuclear gene encoding *Pf*Exo, a mtDNA fragment (mt-F), and an apicoplast DNA sequence encoding Rpl16 were used to amplify nuclear, mitochondrial, and apicoplast DNA, respectively. Primer sequences are detailed in reference 9. The PCR products were electrophoresed on 1% agarose gel.

### Generation of *Pb*Lig1 transgenic parasites and expression analysis

For endogenous tagging of *Pb*Lig1 (PBANKA_1402600) with 3×HA-mCherry, two fragments, F1 (0.6 kb) and F2 (0.61 kb), were amplified using primers 1815/1816 and 1893/1894 and cloned into the pBC-3×HA-mCherry-hDHFR vector at the XhoI/BglII and NotI/AscI sites, respectively. The final construct was linearized with XhoI/AscI, transfected into *P. berghei* ANKA schizonts, and injected into Swiss albino mice as described previously (44). Drug-resistant parasites were selected via positive selection with pyrimethamine. Genomic DNA was isolated from resistant parasites, and 5′ and 3′ site-specific integration was confirmed via diagnostic PCR using primer sets 1963/1392 and 1215/1697, respectively (primer sequences are listed in Table S1 at https://doi.org/10.6084/m9.figshare.30461318). To observe live mCherry expression in the transgenic parasites, iRBCs or mosquito-stage parasites were placed on a glass slide, mounted with a coverslip, and observed under a fluorescence microscope as previously described (45).

### Western blotting

Parasite-infected blood was collected, washed with 1× PBS, lysed with 0.15% saponin, and washed again two to three times with 1× PBS. The parasite pellet was lysed with RIPA buffer, mixed with 5× Laemmli buffer, resolved via 7.5% SDS-PAGE, and transferred to a nitrocellulose membrane. The blot was probed with an anti-mCherry antibody (diluted 1:1,000, Thermo Fisher, Cat. 1C51). The signals were detected using HRP-conjugated anti-rabbit (diluted 1:5,000) (Amersham Biosciences, Cat. NA934V) or anti-mouse IgG (diluted 1:5,000) (Amersham Biosciences, Cat. NA931V) and visualized via a ChemiDoc XRS+ System (Bio-Rad, USA).

### Generation of *Pb*Lig1 KO parasites

To disrupt the gene via the conventional method, a Lig1-targeting plasmid was obtained from Plasmogem. The plasmid was linearized with NotI and transfected into *P. berghei* ANKA schizonts, as previously described (44). Two independent attempts to delete the gene were unsuccessful. Next, we switched to the Flp/FRT-based conditional mutagenesis system to analyze the role of *Pb*Lig1 during preerythrocytic stages, as previously described (35). The targeting plasmid was constructed by amplifying three fragments, F3 (0.1 kb), F4 (2.6 kb), and F5 (0.54 kb), via the primers 1698/1699, 1700/1701, and 1702/1703, respectively, and cloning them into the p3′TRAP-flitre-hDHFR plasmid at the SacII/NotI, EcoRV, and PstI/KpnI sites, respectively. The plasmid was linearized via SacII/KpnI and transfected into *P. berghei* ANKA UIS4/Flp schizonts. We also attempted another strategy by flirting the 3′UTR of the gene (46). In this strategy, two fragments, F6 (0.57 kb) and F7 (0.56 kb), were amplified using primers 1694/2076 and 2077/2078 and cloned into p3′TRAP-flitre-hDHFR-GFP at XhoI/NotI and AscI/XhoI, respectively. The plasmid was linearized via XhoI digestion and transfected into *P. berghei* ANKA UIS4/Flp schizonts. In the third strategy, three fragments, F8 (1.0 kb), F9 (3.15 kb), and F10 (0.51 kb), were amplified via the primers 1698/1699, 2179/2180, and 2181/2182, which were subsequently cloned into a p3′TRAP-flitre-hDHFR plasmid at SacII/NotI, EcoRV, and PstI/KpnI, respectively. The plasmid was linearized via SacII/KpnI and transfected into *P. berghei* ANKA UIS4/Flp schizonts. Transfected parasites were selected via the oral administration of pyrimethamine. Genomic DNA was isolated from the drug-resistant parasites, and correct 5′ and 3′ integration was confirmed using primers 2214/1216 and 1215/2215, respectively. The integration of the FRT site was confirmed via PCR using the primer pair 2216/2217, followed by XbaI digestion. Clonal lines of the conditional KO parasite were obtained by limiting dilutions of the parasites.

### Asexual blood-stage propagation

To analyze the asexual blood-stage propagation of *Pb*Lig1 cKO parasites, an equal number of UIS4/Flp and *Pb*Lig1 cKO parasites were injected i.v. into Swiss albino mice. The progression of parasitemia was monitored daily via Giemsa-stained blood smears.

### Analysis of parasite development in the mosquito

Parasites were transmitted to mosquitoes by allowing them to probe for a blood meal on infected Swiss albino mice. On day 14, post-blood meal, the mosquitoes were dissected to obtain midguts, and the oocysts and sporogony were observed. To enumerate the sporozoite numbers, the midguts were crushed, and the sporozoites were purified and counted using a hemocytometer as previously described (47). On day 17 post-blood meal, the infected cages were shifted to an environmental chamber maintained at 21°C to achieve optimal Flp excision efficiency (48). Salivary glands were dissected on days 20–22 post-blood meal, and the sporozoite numbers were determined as described above. To check the excised flirted locus, genomic DNA was isolated from sporozoites and genotyped using primers 2216/2215.

### *In vivo* infectivity of sporozoite

Salivary gland sporozoites were i.v. injected into C57BL/6 mice (5 mice/group). The appearance of parasites in the blood was monitored via Giemsa-stained blood smears. To estimate the liver-stage parasite burden, another group of C57BL/6 mice was inoculated with 5,000 sporozoites, and the liver was harvested at 36 and 55 hpi in RNAiso Plus reagent (Takara, #9108), and total RNA was isolated according to the manufacturer’s instructions. cDNA was synthesized from 1 µg of RNA, and *Pb*18s rRNA and mouse GAPDH transcripts were amplified via the primer pairs 1195/1196 and 1193/1194, respectively, and quantified using SYBR Green reagent (Takara, #RR420A) (49) in a CFX Opus 96 real-time PCR system (Bio-Rad).

### *In vitro* infectivity of sporozoites

Salivary gland sporozoites were added to the HepG2 culture as previously described (50). Briefly, 55,000 cells/well were seeded in 48-well plates, and after 24 h, 5,000 sporozoites/well were added, after which the cultures were fixed with 4% PFA at different time points.

### IFA

To check the localization of *Pb*Lig1, 3×HA-mCherry-tagged blood-stage parasites were stained with MitoTracker dye (Invitrogen, Cat. M7514) as previously described (51). Blood-stage parasites or sporozoite spots were fixed with 4% PFA for 20 min at RT, washed twice with 1× PBS, and then permeabilized with 0.1% Triton X-100 (Sigma-Aldrich, Cat. T8787) for 10 min at RT. The fixed HepG2 culture was permeabilized with chilled methanol for 20 min at 4°C. The samples were blocked with 1% BSA/PBS for 1 h at RT, followed by incubation with various primary antibodies for 1–2 h at RT. The primary antibodies used were anti-mCherry (diluted 1:1,000; rabbit polyclonal; Abcam, Cat. ab167453), anti-HSP70 (52) (diluted 1:1,000, mouse monoclonal), anti-g377 (53) (diluted 1:100, rabbit polyclonal), anti-tubulin (53) (diluted 1:100, rat polyclonal), anti-CSP (54) (diluted 1:1,000; mouse monoclonal), anti-ACP (55) (diluted 1:1,000, rabbit polyclonal), anti-UIS4 (56) (upregulated in infectious sporozoites 4) (diluted 1:1,000, rabbit polyclonal), and anti-MSP1 (57) (diluted 1:5,000, mouse monoclonal). After washing in PBS, the secondary antibodies, Alexa Fluor 594-conjugated anti-rabbit IgG (diluted 1:1,000; Invitrogen, Cat. A21442), Alexa Fluor 488-conjugated anti-rat IgG (diluted 1:1,000; Invitrogen, Cat. A11006) and Alexa Fluor 488-conjugated anti-mouse IgG (diluted 1:1,000; Invitrogen, Cat. A11001) were used. The slides were washed with PBS, and the nuclei were stained with Hoechst 33342 (Invitrogen, Cat. 62249) and mounted with Diamond antifade reagent (Invitrogen, #P36970). Images were acquired via FV1000 software via a confocal laser scanning microscope (Olympus BX61WI) with a UPlanSAPO 100× (NA 1.4, oil). The images were processed via imageJ software (58). Fluorescence intensity line profiles were generated using ImageJ. Colocalization was inferred from overlapping peaks in the intensity traces of fluorescence signals along the line.

### Statistical analysis

Statistical analysis was performed using GraphPad Prism 9 software. The data are presented as the mean ± SEM or mean ± SD. The statistical significance of differences between groups was analyzed using an unpaired two-tailed Student’s *t*-test or one-way ANOVA.
